# Delayed Minocycline Treatment Ameliorates Hydrocephalus Development and Choroid Plexus Inflammation in Spontaneously Hypertensive Rats

**DOI:** 10.3390/ijms23042306

**Published:** 2022-02-19

**Authors:** Xiaodi Hao, Fenghui Ye, Katherine G. Holste, Ya Hua, Hugh J. L. Garton, Richard F. Keep, Guohua Xi

**Affiliations:** Department of Neurosurgery, University of Michigan, Ann Arbor, MI 48109, USA; haoxiaodi6601@gmail.com (X.H.); fenghuiy@umich.edu (F.Y.); holsteka@umich.edu (K.G.H.); yahua@umich.edu (Y.H.); hgarton@umich.edu (H.J.L.G.); rkeep@umich.edu (R.F.K.)

**Keywords:** hydrocephalus, spontaneously hypertensive rats, minocycline, epiplexus cells, macrophages, choroid plexus, cognitive function

## Abstract

Hydrocephalus is a complicated disorder that affects both adult and pediatric populations. The mechanism of hydrocephalus development, especially when there is no mass lesion present causing an obstructive, is poorly understood. Prior studies have demonstrated that spontaneously hypertensive rats (SHRs) develop hydrocephalus by week 7, which was attenuated with minocycline. The aim of this study was to determine sex differences in hydrocephalus development and to examine the effect of minocycline administration after hydrocephalus onset. Male and female Wistar–Kyoto rats (WKYs) and SHRs underwent magnetic resonance imaging at weeks 7 and 9 to determine ventricular volume. Choroid plexus epiplexus cell activation, cognitive deficits, white matter atrophy, and hippocampal neuronal loss were examined at week 9. In the second phase of the experiment, male SHRs (7 weeks old) were treated with either saline or minocycline (20 mg/kg) for 14 days, and similar radiologic, histologic, and behavior tests were performed. Hydrocephalus was present at week 7 and increased at week 9 in both male and female SHRs, which was associated with greater epiplexus cell activation than WKYs. Male SHRs had greater ventricular volume and epiplexus cell activation compared to female SHRs. Minocycline administration improved cognitive function, white matter atrophy, and hippocampal neuronal cell loss. In conclusion, while both male and female SHRs developed hydrocephalus and epiplexus cell activation by week 9, it was more severe in males. Delayed minocycline treatment alleviated hydrocephalus, epiplexus macrophage activation, brain pathology, and cognitive impairment in male SHRs.

## 1. Introduction

Hydrocephalus is a complicated neurological disorder affecting diverse populations. For example, hydrocephalus occurs in approximately 55% of adult patients with intraventricular hemorrhage, which is an independent indicator of worsened outcomes in acute intracerebral hemorrhage [1]. Hydrocephalus is also a significant cause of morbidity and mortality in the pediatric population with a prevalence of approximately 6 in 10,000 live births [2]. However, the mechanism of hydrocephalus development is still largely unclear, and effective treatment approaches are quite limited [1,3]. While spontaneously hypertensive rats (SHRs) are known to develop stable chronic high blood pressure after birth progressively, they also begin to develop hydrocephalus between 4 and 8 weeks after birth [4] and gradually exhibit poor learning and memory abilities from 5 weeks over to 18 months of age [5,6,7,8]. In a recent study, SHRs were used to establish a spontaneous hydrocephalus model [9].

The mechanisms underlying hydrocephalus in SHRs are still uncertain. However, activation of a group of apically located choroid plexus macrophages, called epiplexus cells, was associated with hydrocephalus development in SHRs [9]. Those cells were also activated in subarachnoid hemorrhage and intraventricular hemorrhage models and more so in rats who went on to develop hydrocephalus [10,11,12].

Minocycline is a broad-spectrum tetracycline with anti-inflammatory actions. It is thought to reduce hydrocephalus in animal models by attenuating reactive gliosis [13,14] and iron overload [15]. Furthermore, administration of minocycline before the onset of hydrocephalus development (i.e., prophylactic treatment) in SHRs ameliorated the hydrocephalus [9]. However, to better mimic actual clinical settings, the current study aimed to identify whether delayed treatment with minocycline could ameliorate hydrocephalus and subsequent brain impairment in SHRs, i.e., after hydrocephalus development has already started and whether this was associated with reduced choroid plexus inflammation.

## 2. Results

### 2.1. Spontaneous Hydrocephalus Developed in SHRs

**Figure 1:**
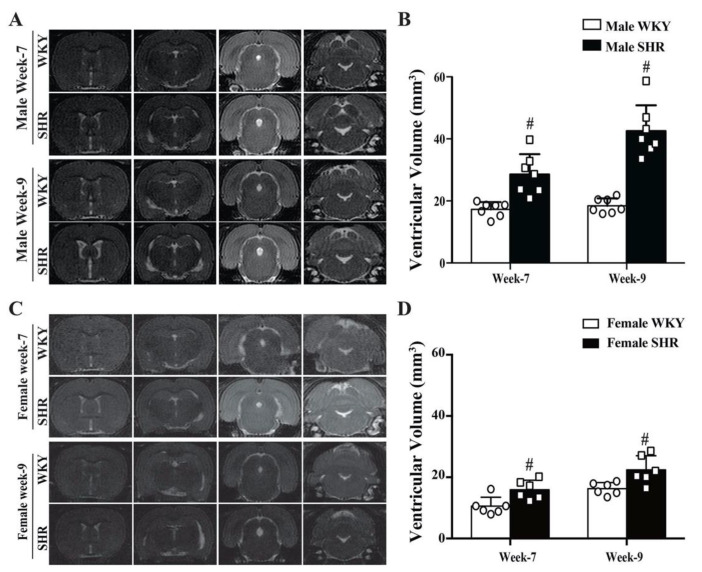
(**A**) T2-weighted MRI images showing ventricular enlargement in male SHRs compared to age-matched WKYs at weeks 7 and 9. (**B**) Quantification of the ventricular volume of male SHRs and WKYs. Values are the means ± SD, *n* = 7; # *p* < 0.01 compared with the male WKY group. (**C**) T2-weighted MRI images showing the ventricular volume in female SHRs and age-matched WKYs at weeks 7 and 9. (**D**) Quantification of the ventricular volume of female SHRs and WKYs. Values are the means ± SD, *n* = 6; # *p* < 0.01 compared with the female WKY group.

Significant hydrocephalus occurred in both male and female SHRs at week 7. The ventricular volume was 29 ± 7 mm^3^ in male SHRs vs. 17 ± 2 mm^3^ in male WKYs (*n* = 7, *p* < 0.01, Figure 1A,B). Similarly, the ventricular volume was 16 ± 3 mm^3^ in female SHRs vs. 11 ± 3 mm^3^ in female WKYs (*n* = 6, *p* < 0.05, Figure 1C,D). Female SHRs developed less ventriculomegaly than male SHRs (*p* < 0.01). The hydrocephalus worsened at week 9 in both male SHRs (43 ± 8 vs. 18 ± 2 mm^3^ in male WKYs, *n* = 7, *p* < 0.01, Figure 1A,B) and female SHRs (22 ± 5 vs. 16 ± 2 mm^3^ in female WKYs, *n* = 6, *p* < 0.01, Figure 1C,D). However, again female SHRs had less severe ventriculomegaly than male SHRs (*p* < 0.01). Dilation of the aqueduct and 4th ventricles implied non-obstructive hydrocephalus.

Male SHRs had significantly higher systolic blood pressures (153.4 ± 9.0 vs. 115.9 ± 7.1 mmHg at week 7, *p* < 0.01; 168.0 ± 15.3 vs. 120.9 ± 3.4 mmHg at week 9, *p* < 0.01) and mean blood pressure (125.6 ± 9.4 vs. 91.1 ± 6.4 mmHg at week 7, *p* < 0.01; 135.4 ± 13.9 vs. 96.6 ± 1.3 mmHg at week 9, *p* < 0.01) compared with that in male WKYs. The blood pressure of female SHRs was also significantly higher than that of female WKYs in both systolic blood pressure (153.3 ± 19.5 vs. 115.8 ± 5.7 mmHg at week 7, *p* < 0.01; 151.8 ± 6.1 vs. 122.3 ± 6.8 mmHg at week 9, *p* < 0.01) and mean blood pressure (133.5 ± 17.4 vs. 91.2 ± 6.3 mmHg at week 7, *p* < 0.01; 130.1 ± 9.43 vs. 99.4 ± 6.4 mmHg at week 9, *p* < 0.01).

### 2.2. Activation of Epiplexus Macrophages in SHRs 

**Figure 2:**
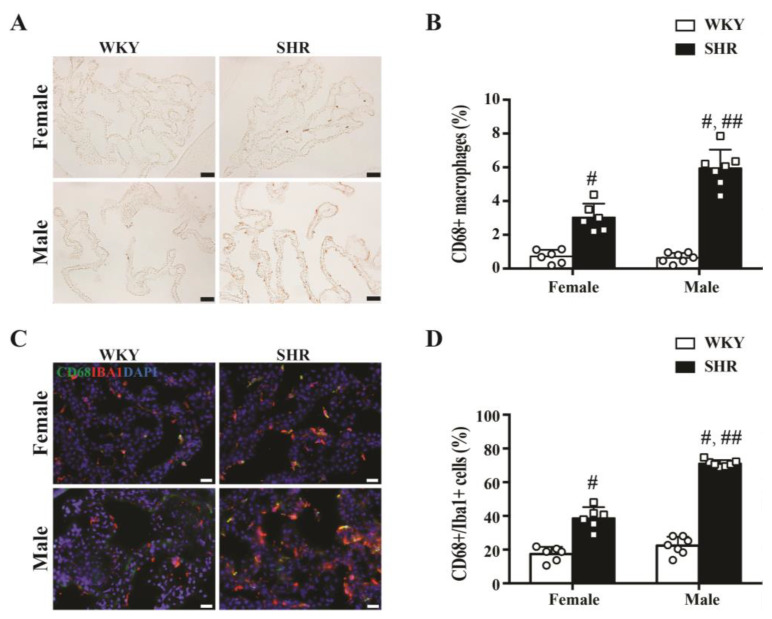
(**A**) Example of CD68 immunoreactivity in the choroid plexus of male and female SHRs and WKYs at week 9 (scale bar = 50 μm). (**B**) CD68-positive cells as a percentage of all choroid plexus cells in male and female SHRs and WKYs at week 9. Values are the means ± SD, *n* = 6−7; # *p* < 0.01 compared with the WKY groups. Male and female SHRs had more CD68+ cells than WKYs. In addition, male SHRs had more CD68-positive cells than female SHRs; ## *p* < 0.01 compared with the female SHR group. (**C**) Co-localization of CD68- and Iba1-positive cells at the choroid plexus in male and female rats at week 9 (scale bar = 20 μm). (**D**) Percentage of Iba-positive cells that were also positive for CD68 at the choroid plexus. Values are the means ± SD, *n* = 6–7; # *p* < 0.01compared with the WKY groups. In both male and female SHRs, a greater % of Iba+ cells were CD68+ than in WKYs. In addition, male SHRs had a greater % than female SHRs; ## *p* < 0.01 compared with the female SHR group.

CD68 was used as a marker of activated macrophages in this study. The number of CD68-positive cells in the choroid plexus (expressed as a % of total cells) was significantly higher in SHRs compared to WKYs at week 9 in both males (5.9 ± 1.1 vs. 0.6 ± 0.3%, *n* = 7, *p* < 0.01) and females (3.0 ± 0.8 vs. 0.7 ± 0.4%, *n* = 6, *p* < 0.01, Figure 2A,B). In addition, 71 ± 2% of Iba1-positive cells were also CD68-positive in male SHRs, compared with 22 ± 5% of male WKYs at week 9 (*n* = 7, *p* < 0.01, Figure 2C,D), while 39 ± 6% of Iba1-positive cells were CD68-positive in female SHRs compared to 17 ± 4% in female WKYs at week 9 (*n* = 6, *p* < 0.01, Figure 2C,D). A significant difference between male and female SHRs in macrophage activation at the choroid plexus was also observed, both in terms of the number of CD68-positive cells (*p* < 0.01, Figure 2B) and the degree of CD68+/Iba1+ co-localization (*p* < 0.01, Figure 2D). 

### 2.3. Cognitive Function and Neuronal Loss in Male SHRs

**Figure 3:**
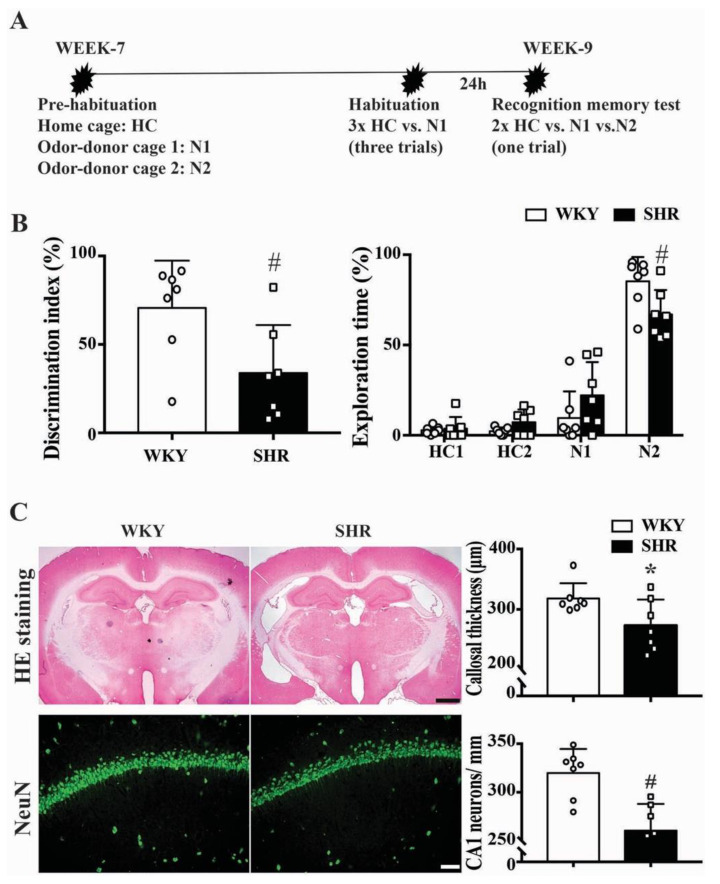
(**A**) Representation of the experimental procedure. HC = home cage bead; N1 = familiar odor bead; N2 = unfamiliar novel odor bead. (**B**) Bar graphs showing the discrimination index ratio (%) and the exploration time on the N2 bead in at week 9 in male WKYs and SHRs. Values are the means ± SD, *n* = 7; # *p* < 0.01 compared with the WKY group. (**C**) Representative H&E stained coronal sections from WKYs and SHRs at week 9 (scale bar = 1 mm) that were used to determine corpus callosum thickness, and representative NeuN immunoreactivity in the hippocampus CA1 area (scale bar = 50 μm) that was used to determine neuronal loss. Quantification of the results are shown in the bar graphs. Values are the means ± SD, *n* = 7; * *p* < 0.05, # *p* < 0.01.

With a novel object recognition test (Figure 3A), a significant difference in the discrimination index (34 ± 27 vs. 71 ± 27%, *p* < 0.05) and the time spent exploring a novel bead (67 ± 14 vs. 85 ± 13%, *n* = 7, *p* = 0.0251) was noted between male SHRs and male WKYs at week 9 (Figure 3B). Brain histology showed corpus callosum and hippocampus atrophy in SHRs at week 9. SHRs had reduced corpus callosum width compared to WKYs (273 ± 43 vs. 318 ± 25 μm, *n* = 7, *p* < 0.05, Figure 3C). In addition, SHRs had fewer hippocampal CA1 neurons compared with WKYs (261 ± 28 vs. 320 ± 25 CA1 neurons/mm, *n* = 7, *p* < 0.01, Figure 3C).

### 2.4. Hydrocephalus and Blood Pressure after Minocycline Treatment in Male SHRs

**Figure 4:**
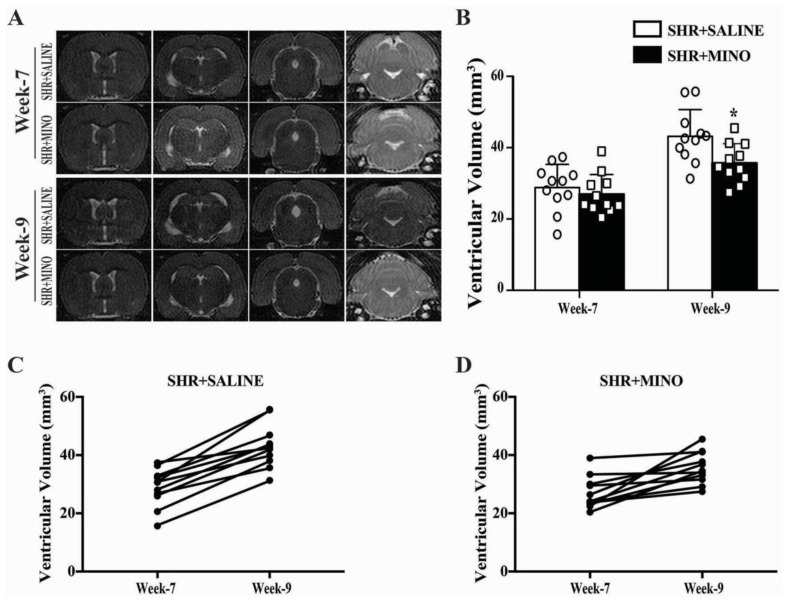
(**A**) Representative T2 images of male SHRs treated with saline or 20 mg/kg/12 h minocycline (MINO) for 14 days. Images were taken prior to treatment (week 7) and after treatment (week 9). (**B**) The images were used to quantify ventricular volume at both time points. Values are the means ± SD, *n* = 11; * *p* < 0.05, compared with the SHR + saline group. (**C**,**D**) Tracking of changes in ventricular volume for individual rats between weeks 7 and 9.

Fourteen days of minocycline (20 mg/kg/12 h) treatment starting from week 7 attenuated ventricular dilation on T2 images compared to saline treatment in male SHRs (36 ± 5 vs. 43 ± 7 mm^3^, *n* = 11, *p* < 0.05, Figure 4A,B). The individual ventricular volume enlargement between week 7 and week 9 significantly decreased in the minocycline treatment group (9 ± 7 mm^3^) compared with that in the vehicle treatment group (14 ± 6 mm^3^, *n* = 11, *p* < 0.05, Figure 4C,D).

The baseline (week 7) blood pressure in the minocycline and vehicle groups were not significantly different (systolic blood pressure: 156 ± 6 vs. 162 ± 14 mmHg; mean blood pressure: 132 ± 14 vs. 126 ± 6 mmHg). However, minocycline treatment lowered systolic blood pressure at week 8 (150 ± 7 vs. 164 ± 10 mmHg, *p* < 0.01) and week 9 (148 ± 10 vs. 167 ± 12 mmHg, *p* < 0.01) and the mean blood pressure at week 8 (133 ± 10 vs. 120 ± 8 mmHg, *p* < 0.01) and week 9 (135 ± 13 vs. 117 ± 10 mmHg, *p* < 0.01).

### 2.5. Cognitive Function and Neuron Loss after Minocycline Treatment

**Figure 5:**
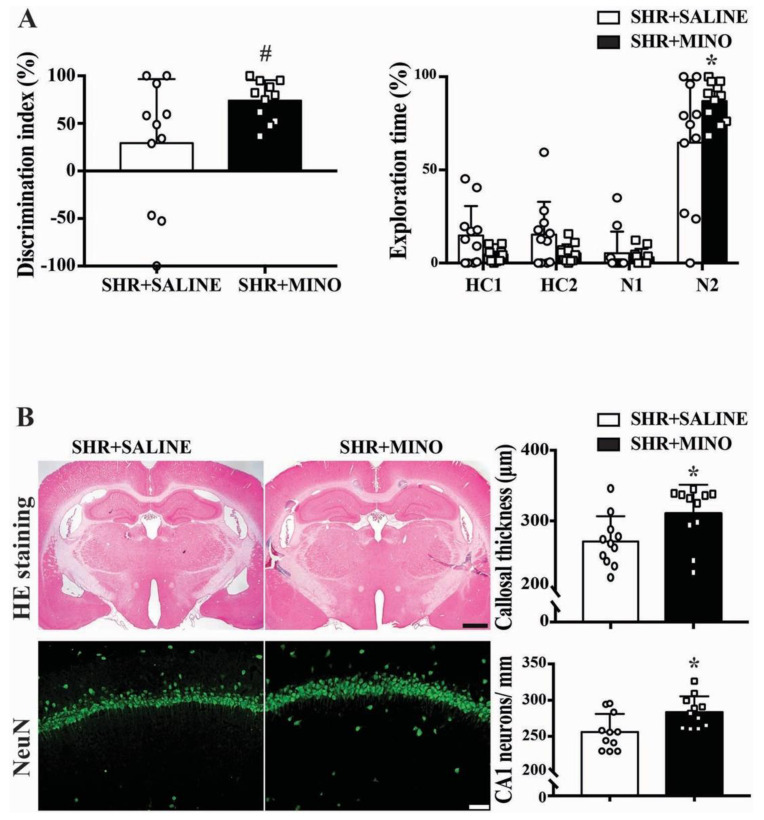
(**A**) Bar graphs showing the discrimination index and the exploration time on N2 beads in male SHRs treated with minocycline (MINO) or saline. Values are the means ± SD, *n* = 11; * *p* < 0.05, # *p* < 0.01. (**B**) Representative H&E stained coronal sections (scale bar = 1 mm) and NeuN immunoreactivity in the CA1 region of the hippocampus (scale bar = 50 μm) in male SHRs treated with minocycline or vehicle at week 9. The H&E sections were used to determine the corpus callosum thickness and the NeuN staining the number of neurons/mm. Values are the means ± SD, *n* = 11; * *p* < 0.05.

With minocycline treatment, male SHRs exhibited better time spent exploring a novel bead (86.97 ± 10.7 vs. 64.65 ± 33.64%, *n* = 11, *p* < 0.05) and discrimination index (73.95 ± 21.4 vs. 29.29 ± 67.28%, *n* = 11, *p* < 0.05) at week 9 (Figure 5A).

H&E staining showed increased corpus callosum thickness (310.5 ± 40.8 vs. 270.8 ± 35.8 μm, *p* < 0.05,) and more CA1 neurons in the hippocampus (283.7 ± 21.9 vs. 256.1 ± 25.1 neurons/mm, *p* < 0.05) in the minocycline treatment group at week 9 (Figure 5B).

### 2.6. Activation of Epiplexus Macrophages after Minocycline Treatment

**Figure 6:**
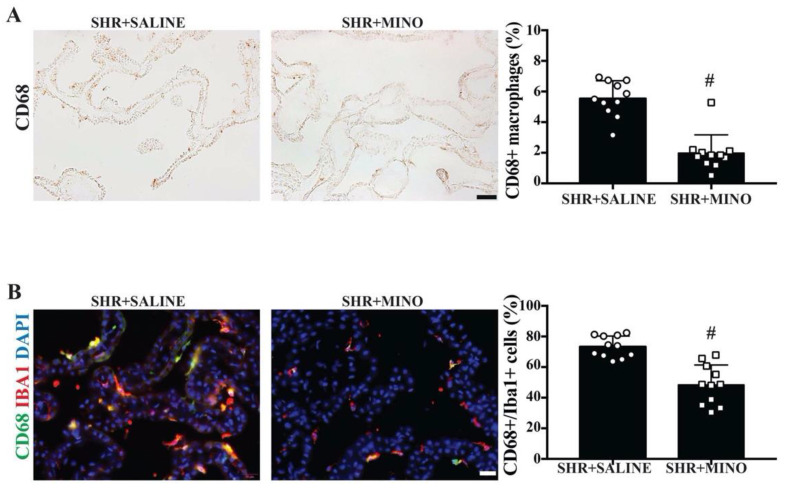
(**A**) Examples of CD68 immunoreactivity at the choroid plexus in male SHRs treated with saline or minocycline (MINO) at week 9 (scale bar = 50 μm) along with quantification. The number of CD68-positive cells was expressed as a percentage of all choroid plexus cells. (**B**) Examples of double-labeling immunofluorescence of the choroid plexus examining CD68 and Iba1 co-localization in epiplexus cells along with DAPI to identify cell nuclei (scale bar = 20 μm). The percentage of Iba+ cells that were also CD68-positive was quantified for SHRs treated with saline and minocycline. For both (**A**) and (**B**), values are the means ± SD, *n* = 11; # *p* < 0.01 between the saline and minocycline groups.

Minocycline treatment reduced the number of activated macrophages (CD68+) in the choroid plexus of SHRs at week 9 compared with the vehicle group (2.0 ± 1.2 vs. 5.5 ± 1.2% of total choroid plexus cells, *n* = 11, *p* < 0.01, Figure 6A). In addition, the percentage of Iba+ macrophages that were also CD68-positive was significantly reduced by minocycline treatment (48 ± 13 vs. 73 ± 7% in the vehicle group, *p* < 0.01, Figure 6B).

## 3. Discussion

The major findings of this study were that (1) both male and female SHRs developed hydrocephalus at week 7, which worsened by week 9; (2) male SHRs had larger ventricular volumes than their female counterparts at weeks 7 and 9; (3) in both male and female SHRs, there was greater epiplexus (macrophage) cell activation at the choroid plexus than in WKYs at week 9, and that activation was also greater in male SHRs than in females. Due to the fact of this significant difference between male and female SHRs, the rest of the study focused on male SHRs only; (4) treatment with minocycline after the onset of hydrocephalus development reduced further ventricular enlargement; (5) nine-week-old male SHRs developed cognitive deficits, corpus callosum thinning, and hippocampal neuronal loss that were attenuated by delayed minocycline treatment; (6) delayed minocycline treatment also ameliorated the choroid plexus inflammatory response with reduced epiplexus cell activation.

Sex differences are an important topic for both clinical and preclinical neurological studies. In the setting of ischemic stroke, it is well known that sex influences many variables including cerebrovascular anatomy, pathological changes, therapeutic reactions, and prognosis [16,17]. However, research on sex differences in hydrocephalus has been quite limited. Our previous study indicated that male SHRs developed significant hydrocephalus at 7 weeks [9]. The present study found that female SHRs also presented with hydrocephalus at 7 weeks. However, this was less severe than the male SHRs and, while both sexes had progressive hydrocephalus at 9 weeks old, this effect was reduced in females. Female SHRs exhibited less choroid plexus inflammation (epiplexus cell activation) than males. Estrogen has strong anti-inflammatory properties and might affect epiplexus cell activation, because the female rats were not ovariectomized [18,19]. As such, these data indicate that sex may influence the development of hydrocephalus in SHRs. We also found a statistically significant enlargement in ventricular volume in female WKYs from week 7 to week 9. It is still unclear why ventricle volumes increased from week 7 to week 9 in female WKYs rather than in males. Although spontaneous hydrocephalus has been reported in female Wistar rats [20], in the current study, ventricle volumes in 9 week old female WKYs were the same as in males (less than 20 mm^3^). It should be noted that sex differences in hydrocephalus may be disease specific. For example, hydrocephalus after subarachnoid hemorrhage is more severe in females [21]. 

Hydrocephalus is classified as obstructive or non-obstructive hydrocephalus. In obstructive hydrocephalus, the etiology is thought to be due to the obstructive lesion causing reduced flow and absorption of cerebrospinal fluid (CSF) throughout the ventricular system. In non-obstructive hydrocephalus, on the other hand, CSF absorption is still reduced, but there is no obstructive lesion present that causes enlargement of all four ventricles. The etiology of non-obstructive hydrocephalus is much less well understood [22]. The dilation of the aqueduct and fourth ventricle in the SHRs suggested non-obstructive hydrocephalus. The mechanisms of hydrocephalus development in SHRs, which present with featured hypertension since birth [23], remain largely unknown. Antihypertensive treatment successfully reduces blood pressure but fails to attenuate the ventriculomegaly seen in SHRs [23]. Additionally, female SHRs in this study were less likely to demonstrate hydrocephalus compared to male counterparts even though their blood pressures were similar. These data suggest that there is some other underlying etiology for hydrocephalus development in these animals. Persistent hypertension can induce changes in the choroid plexus that may affect the brain stroma, blood vessels, and CSF production. These effects were more remarkable for the blood–CSF barrier than for the blood–brain barrier [24,25], indicating that persistent hypertension could cause a significant imbalance between CSF production and absorption in these animals. 

In the present study, a novel object recognition task was employed to detect cognitive deficits. There was a significant difference in the discrimination index and the time spent exploring a novel object between male SHRs and male WKYs. Arterial hypertension can induce the progressive remodeling of brain vessels and lead to constant cognitive decline [26]. SHRs are usually used as a model of cerebral small vessel disease (cSVD) [8,27,28] or attention-deficit hyperactivity disorder (ADHD) disease [5,29,30], which are related to stable brain impairment [31,32,33,34]. In the current study, histology staining showed marked corpus callosum atrophy and hippocampal neuronal loss in SHRs, which may explain the demonstrated cognitive function deficits. The possible relationship between the cerebral small vessel disease and the hydrocephalus in SHRs needs further investigation.

CSF secretion by the choroid plexuses can be induced by inflammation [35]. Cell immunity and neuroinflammation have been widely discussed in hydrocephalus pathogenesis [36,37,38]. Our previous study suggested that the hydrocephalus in SHRs was related to epiplexus cell activation [9]. CD68, as a marker of activated macrophages, has been used to indicate macrophage participation in hydrocephalus development [35,39,40]. The current study found more CD68-positive cells at the choroid plexus in SHRs compared to WKYs at week 9 in both male and female SHRs. However, the male SHRs had greater macrophage activation compared with female SHRs, which was consistent with the difference in the ventricular volumes of those two sexes. Prior studies suggest that activated macrophages produce proinflammatory cytokines, such as interleukin-1β, which could participate in the impairment of ependymal cilia formation [41] and stimulate Na–K–Cl cotransporter-1 at the choroid plexus epithelium, increasing CSF secretion [35] and, subsequently, contributing to hydrocephalus. The present study further demonstrated the relationship between the choroid plexus inflammation with epiplexus cell activation and hydrocephalus development in male and female SHRs. However, CD68-positive cells observed in the choroid plexus may include brain infiltrating macrophages and resident phagocytic cells. Thus, the origin of those activated CD68-positive cells in the SHRs is uncertain. Future studies may use microglia-specific markers, such as TMEM119, to distinguish brain infiltrating macrophages from cells of microglial lineage.

Minocycline is a broad-spectrum tetracycline that can penetrate the blood–brain barrier. It has anti-inflammatory effects and may be protective in multiple neurological disorders including ischemic or hemorrhagic stroke [42,43,44], multiple sclerosis [45,46], and Parkinson’s disease [46]. The mechanisms underlying minocycline neuroprotective effects may relate to inhibiting macrophage activation [47,48], attenuating iron overload [15,42,43,49], reducing the astrocyte gliosis [50], and preventing demyelination [51]. 

In SHRs, we previously demonstrated that minocycline treatment initiated prior to hydrocephalus development (week 5) could reduce hydrocephalus and epiplexus cell activation in SHRs at week 7 [9]. In order to better mimic clinical settings where patients usually present with preexisting hydrocephalus, this study focused on the effects of delayed minocycline administration in SHRs. Delayed minocycline administration attenuated the progression of ventricular enlargement as well as cognitive function defects and hippocampus neuron loss, i.e., minocycline treatment was beneficial even after the onset of hydrocephalus. The improved performance in the cognitive task with minocycline could be due to the improved corpus callosum thickness and hippocampal neuron loss as seen on histology. Moreover, minocycline reduced an inflammatory phenotype at the choroid plexus (number of CD68-positive epiplexus cells). This is consistent with our previous study concerning the effects of minocycline on epiplexus cell activation [9]. 

This study had several limitations: (1) Only one minocycline dosing regimen was examined (20 mg/kg/12 h for 2 weeks). The optimal dose and therapeutic duration need to be investigated. (2) The exact relationship between epiplexus cell activation and hydrocephalus is still unclear. Thus, is the activation the result or the cause of the hydrocephalus? (3) Although this was a randomized study, researchers were not blinded to experimental conditions. (4) Some behavioral data regarding the discrimination/exploration time might be affected by the rat itself, since SHRs are often used as genetic model of ADHD. (5) Minocycline is not a specific inhibitor of macrophage/microglia. For example, minocycline also acts as an iron chelator. (6) Minocycline reduced epiplexus cell activation and hydrocephalus in SHR rats, but the effects of minocycline on epiplexus cell activation needs to be tested in an IVH model. (7) Only the CA1 region was quantified to determine the neuronal loss of the hippocampus.

In summary, this study demonstrated that both male and female SHRs had hydrocephalus by week 7, which worsened by week 9 and was more severe in males. Epiplexus cell activation occurred at the choroid plexus in SHRs of both sexes but was more extensive in males. Treatment with minocycline after the onset of hydrocephalus (week 7) still ameliorated hydrocephalus progression, cognitive deficits, and hippocampus neuron loss as well as epiplexus cell activation in male SHRs. In combination with our previous results on the prophylactic effect effects of minocycline on hydrocephalus in SHRs, we believe that minocycline could be a promising therapeutic target for some forms of hydrocephalus.

## 4. Materials and Methods

### 4.1. The Animal Preparation 

The University of Michigan Committee on the Use and Care of Animals approved all animals used in the study (PRO00007721, 2 June 2017). A total of 36 male rats and 12 female rats (aged 7 weeks, Charles River Laboratories, Wilmington, MA, USA) were used. Blood pressure was measured using the tail-cuff method (coda 6; Kent Scientific Corporation, Torrington, CT, USA). Systolic and diastolic blood pressure was recorded at weeks 7, 8, and 9, and the mean blood pressure (MBP) was calculated. The study complied with the ARRIVE guidelines for reporting in vivo experiments.

### 4.2. Experimental Groups

The animals were distributed into two parts. First, both male and female SHRs and WKYs aged 7 weeks received magnetic resonance imaging (MRI) scanning at weeks 7 and 9 to determine the ventricle volumes (*n* = 6–7 for each group). Animals were euthanized at week 9 post-MRI after cognitive function determination, and the brains were harvested for histology. Second, male SHRs (7 weeks old) were treated with intraperitoneal minocycline or saline for 14 days (*n* = 11 per group). MRI was performed before and after the treatment, and cognitive function tests were performed at week 9 before euthanasia, and the brains were harvested for histology.

### 4.3. Minocycline Administration

Minocycline was injected intraperitoneally into the male SHRs every 12 h at 20 mg/kg for 14 days starting from week 7. The dose used was based on our previous study to prevent hydrocephalus development in SHRs [9]. Randomization was carried out using odd/even numbers. Rats in the vehicle group received the same volume of saline at the same times. Animals were monitored for adverse reactions such as diarrhea and decreased body weight. 

### 4.4. Magnetic Resonance Imaging (MRI) and Ventricle Volume Measurement

MRI was performed at weeks 7 and 9. Rats were anesthetized with 2% isoflurane and scanned with a 9.4T MRI (Agilent, Palo Alto, CA, USA). A T2 fast spin-echo sequence was performed with the following parameters: a view field of 35 × 35 mm; matrix of 256 × 256; 25 coronal slices; 0.5 mm thickness. Ventricular volume was calculated as previously described [9]. Briefly, bilateral ventricles were delineated on each slice throughout the entire ventricle distribution using ImageJ software (NIH, Bethesda, MD, USA) to obtain the ventricular area. Ventricular volume was calculated by summing the ventricle area on each slice and multiplying by slice thickness.

### 4.5. Immunohistochemistry and Hematoxylin and Eosin (HE) Staining

Rats were anesthetized with pentobarbital and underwent transcardiac perfusion with 4% paraformaldehyde in 0.1 M PBS (pH 7.4). Brains were removed and placed in paraformaldehyde for 24 h before being transferred to 30% sucrose in 0.1 M PBS at 4 °C for 3 days. After embedding the brains in a mixture of 30% sucrose and optimal cutting temperature compound (Sakura Finetek, Inc., Torrance, CA, USA), 18 μm frozen sections were obtained for brain histology. The primary antibodies were mouse anti-CD68 (1:100 dilution; Abcam, Cambridge, MA, USA), goat anti-Iba1 (1:200 dilution; Abcam, Cambridge, MA, USA), and rabbit anti-NeuN (1:200 dilution; Abcam, Cambridge, MA, USA). The secondary antibodies were horse anti-mouse IgG (1:500 dilution; Vector Laboratories, Burlingame, CA, USA), Alexa Fluor 488 donkey anti-mouse IgG (H+L) (1:500 dilution; Invitrogen, Eugene, OR, USA), Alexa Fluor 594 donkey anti-goat IgG (1:500 dilution; Invitrogen, Eugene, OR, USA), and Alexa Fluor 488 donkey anti-rabbit IgG (H+L) (1:500 dilution; Invitrogen, Eugene, OR, USA). Fluoroshield with DAPI (F6057) was used for nuclear labeling. The negative control was performed by eliminating the primary antibody. CD68-positive cells are presented as a percentage of all choroid plexus cells and CD68+/Iba1+ co-localization is presented as a percentage of all Iba1-positive cells. H&E staining was performed as previously described. For callosal thickness measurements, brain sections at Bregma −3.6 mm were used.

### 4.6. Novel Object Recognition Test

A novel object recognition test was used to evaluate the cognitive function of the rats [52]. The test included three steps as previously reported [53,54]. (1) Habituation phase: In preparation for the odor recognition task, several 2.5 cm diameter round wooden beads with a 0.2 cm hole bored through their diameter (Woodworks Ltd.; Haltom City, TX, USA) were labeled with a unique identifier. Four clean wooden beads were placed in the cages of the test rats (home cage (HC) beads) or odor donor (OD) rats to acquire the odor of the animal and serve as familiar or novel odors in subsequent tests. Beads were introduced into the cages at week 7 and kept to week 9 to build-up animal-specific odors.

(2) Familiarization phase: The four HC beads were removed 1 h before testing the rat. After this 1 h period, a novel-odor wood bead (N1), taken from an odor-donor cage, and three HC beads were introduced into a new clean cage. Rats were exposed to these four beads for three 1 min trials with 1 min intervals. For each 1 min trial, the spatial arrangements of the four beads were randomly altered to avoid location effects. The timing for the 1 min trial began with the initiation of olfactory exploration of any bead. During the trials, exploration time for each of the four beads, defined as sniffing, licking, chewing, or having moving vibrissae while directing the nose toward and <1 cm from the object, was timed separately.

(3) Test phase: Twenty-four hours after the novel-odor habituation phase, rats were tested for their ability to recognize the novel odor that they were exposed to the day before. For the test, each rat was presented with two HC beads: one odor N1 bead taken from the same cage as in the habituation phase and one unfamiliar novel-odor bead (N2) taken from a different odor-donor cage. As in the test phase, the timing of this odor recognition trial began when the rat initiated olfactory exploration of any bead. Only one round of the 1 min trial from when the animal started sniffing any bead was conducted, and the time spent exploring each bead was recorded for the 1 min trial. The discrimination index (DI) was calculated as the time spent exploring the N2 compared with the familiar objects relative to the total time spent exploring all objects, according to the formula [55]:

(1) $$\mathrm{DI}=\frac{\left(t\;\left[N2\right]-t\;\left[\mathrm{familiar}\right]\right)}{\left(t\;\left[N2\right]+t\;\left[\mathrm{familiar}\right]\right)}\times 100\%$$

### 4.7. Statistical Analysis

All data are presented as the mean ± SD. Differences between groups were analyzed by the Student’s *t*-tests and two-way ANOVA with a Bonferroni multiple comparisons test. Statistical significance between groups was considered when *p* < 0.05.

## Data Availability

All supporting data are available within the article.
